# Microemulsion-Based Media in Nose-to-Brain Drug Delivery

**DOI:** 10.3390/pharmaceutics13020201

**Published:** 2021-02-02

**Authors:** Anna Froelich, Tomasz Osmałek, Barbara Jadach, Vinam Puri, Bozena Michniak-Kohn

**Affiliations:** 1 Chair and Department of Pharmaceutical Technology, Poznan University of Medical Sciences, 6 Grunwaldzka Street, 60-780 Poznań, Poland; tosmalek@ump.edu.pl (T.O.); bajadach@ump.edu.pl (B.J.); 2 Center for Dermal Research and Ernest Mario School of Pharmacy, Rutgers, The State University of New Jersey, Piscataway, NJ 08854, USA; vp239@dls.rutgers.edu (V.P.); michniak@pharmacy.rutgers.edu (B.M.-K.)

**Keywords:** microemulsion, nose to brain, drug delivery, blood–brain barrier

## Abstract

Nose-to-brain drug delivery has recently attracted enormous attention as an alternative to other delivery routes, including the most popular oral one. Due to the unique anatomical features of the nasal cavity, drugs administered intranasally can be delivered directly to the central nervous system. The most important advantage of this approach is the ability to avoid the blood–brain barrier surrounding the brain and blocking the entry of exogenous substances to the central nervous system. Moreover, selective brain targeting could possibly avoid peripheral side effects of pharmacotherapy. The challenges associated with nose-to-brain drug delivery are mostly due to the small volume of the nasal cavity and insufficient drug absorption from nasal mucosa. These issues could be minimized by using a properly designed drug carrier. Microemulsions as potential drug delivery systems offer good solubilizing properties and the ability to enhance drug permeation through biological membranes. The aim of this review is to summarize the current status of the research focused on microemulsion-based systems for nose-to-brain delivery with special attention to the most extensively investigated neurological and psychiatric conditions, such as neurodegenerative diseases, epilepsy, and schizophrenia.

## 1. Introduction

**Figure 1:**
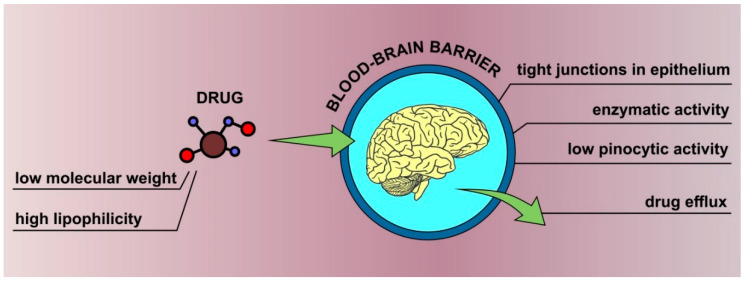
Basic factors affecting the permeability of the blood–brain barrier.

Efficient delivery of active pharmaceutical ingredients (APIs) to the brain is crucial for successful therapy of numerous neurological and psychiatric disorders. In the case of conventional formulations administered orally or parenterally, the drug must cross several biological barriers before it gets into the brain circulation. The most important factor limiting the efficacy of such treatment is the blood–brain barrier (BBB), a unique structure protecting brain from potentially harmful exogenous factors, e.g., chemicals and microbes [1]. The BBB is formed by specialized endothelial cells packed differently than other similar cells in the body. The most important feature determining its properties is the presence of tight junctions in the paracellular space between adjacent cells. These structures consisting of several specific transmembrane proteins, like claudin, occludin, and junction adhesion molecules [2], are essential for limiting the permeability of the BBB to hydrophilic molecules including drugs. This physical barrier between the blood and the central nervous system (CNS) is further supported by an enzymatic barrier, low pinocytic activity, and several drug efflux mechanisms, including P-glycoprotein and other multidrug resistance proteins, responsible for the removal of exogenous substances from the brain circulation [1,3]. As indicated by Pardridge [4], the difficulties encountered in overcoming the barrier are very frequent and should rather be treated as a rule since more than 98% small molecule drugs are not able to cross it, even though low molecular weight not exceeding 400 Da along with high lipophilicity are usually considered as factors favorable for permeation [5]. Taking into account the fact that the BBB is practically impermeable to macromolecular compounds [6], it is considered as the most difficult biological membrane in terms of drug delivery [3]. The most important factors affecting BBB permeability are summarized in Figure 1.

In order to deliver effective amounts of the active ingredients to the brain, several invasive and semi-invasive methods have been proposed in the past [7]. The invasive techniques included direct intracerebral therapies [8] involving a bolus injection or infusion into the parenchymal region of the brain [9]. Another approach involves intracerebral implants releasing the drug in a prolonged manner by using a biodegradable polymer incorporating the drug. A manufactured example is Gliadel™ (Eisai Inc.), which is a polymer wafer with carmustine implanted in the cavity formed after the surgical removal of malignant glioma from the brain [10]. Drugs can be also delivered directly into the cerebrospinal fluid present in the subarachnoid space surrounding the brain and in the central canal of the spinal cord. This approach is known as intrathecal drug administration. All of the described techniques are more or less invasive, which may result in operative and post-operative complications, e.g., hemorrhages, catheter malfunction or malposition, or catheter-related infections [11,12,13].

Less invasive techniques utilized to overcome the problems related to low BBB permeability include its disruption by approaches like application of hyperosmotic agents [14] or ultrasounds [15]. In all methods involving temporary BBB disruption, the importance of reversibility and duration of tight junctions opening must be emphasized in order to maintain both therapeutic efficacy and safety, especially when considering repeating the procedure. It is noteworthy that increasing permeability of BBB to drugs also exposes the brain to potentially harmful exogenous agents [16].

Considering the challenges related to effective drug delivery to the brain and potential side effects of most direct methods, numerous studies have been aimed at the development of novel, safe, and non-invasive methods for the same. The most common approaches focus on chemical modification of the drug to improve its ability to permeate across the BBB. For this purpose, the active ingredient can be chemically bonded to some transport vector forming so called “Trojan horse” carriers enabling drug transport to brain tissue [17,18] as brain-selective vectors such as insulin [19], transferrin [20], and low-density lipoproteins [21] can easily penetrate the barrier by receptor-mediated transport. Peptide-based active ingredients can be transformed into their cationic form, which has the ability to interact with negatively charged structural elements of the BBB [22]. Another approach involves applying inactive prodrugs, which display better ability to penetrate across the tight junctions in epithelium and are transformed into active ingredient at the site of action [23,24]. Moreover, the drugs delivered to the brain tissue can also be encapsulated in various carriers, including cyclodextrins [25,26], liposomes [27,28], and nanoparticulate systems [29,30] in order to improve their ability to cross the BBB. Recently, one of the most extensively investigated non-invasive methods for drug delivery to the central nervous system involves nasal cavity as an administration site. The unquestionable advantages of this route are ease of application and rapid absorption of active ingredients from the nasal mucosa followed by direct transport to the brain without hepatic first-pass effect decreasing its efficacy. It is noteworthy that in this way the BBB is bypassed [1,31]. Therefore, molecular mass of the drug in this case is less important for the absorption process, and it has been reported that both small [32,33] and large molecules [34,35] can be successfully delivered in this way. However, despite numerous interesting features, there are also several drawbacks related to this administration route. Among the most important ones possible, drug degradation in nasal mucosa, limited capacity of the nasal cavity, and its high clearance are mentioned [36]. In order to improve the effectiveness of nose-to-brain drug delivery, different carriers are employed, including mucoadhesive formulations, polymeric [37], and lipid nanoparticles [38], micelles [39], nanostructured lipid carriers [40], nanoemulsions [41], and microemulsions [42].

In this review, we focused on microemulsions as one of the most investigated nanodispersion classes. Microemulsions were described for the first time in 1940s [43] and since then they have been subjected to numerous scientific studies, including investigations aiming at the development of novel carriers for drug delivery [44,45,46,47]. Over the decades, it has been shown that they reveal great potential in terms of increasing bioavailability of active pharmaceutical ingredients, particularly those classified as poorly water-soluble [47,48,49,50,51]. The aim of this study was to summarize available literature reports referring to microemulsions and microemulsion-based media applied as vehicles in nose-to-brain drug delivery and to show potential usefulness of these systems in brain-targeting therapeutic approaches, indicating both their advantages and disadvantages. It is important to note that nose-to-brain drug delivery has become one of the most extensively investigated therapeutic approach in which the proper selection of the carrier is crucial for the observed efficacy of the treatment. Microemulsions can be considered as promising vehicles for different therapeutic agents delivered in this way; however, they are not free from drawbacks and the summarized research also show some questions that have not been properly addressed yet.

## 2. Microemulsions: Structure, Properties, and Applications

### 2.1. Definition and Structure

Microemulsions were described for the first time by Hoar and Schulman [43] who performed an experiment involving coarse emulsions titrated with a co-surfactant. As a result, they observed turbid emulsions transforming spontaneously into transparent, isotropic liquids, which were later described as microemulsions. According to the commonly accepted definition formulated by Danielsson and Lindman [52], a microemulsion is an optically isotropic and thermodynamically stable liquid system composed of water, oil, and an amphiphile, which is a surfactant usually enhanced by a co-surfactant. The definition allows for unambiguous differentiation between microemulsions and other similar colloidal systems, e.g., micellar solutions containing only polar or non-polar phase, coarse emulsions, and nanoemulsions that are not thermodynamically stable or liquid crystals that are not isotropic. It is obvious that the qualitative composition of microemulsion is similar to the components necessary to obtain a coarse emulsion. However, the most significant difference between these systems is the transparency observed in microemulsions. It is important to note that the diameter of the dispersed phase particles usually does not exceed 100 nm, which is much less than the visible light wavelength [53,54]. As a result, the light passing the system does not interact with the dispersed phase particles and is not diffracted.

The term ‘microemulsion’ is sometimes considered misleading as it implies the micrometer range of particle sizes and microemulsions are in fact nanodispersions [55,56]. On the other hand, they display several similarities to nanoemulsions, even though from the thermodynamic point of view they are completely different systems. Both dispersions contain polar and non-polar phases stabilized by one or more surfactants. Moreover, because of small dispersed phase droplets present in nanoemulsions, they are also perceived as transparent or translucent liquids. However, nanoemulsions are only kinetically stable, which means that they occupy a metastable state and can potentially undergo destabilization over time. Nevertheless, destabilization time is usually extended because of physical factors preventing coalescence of the droplets, e.g., steric and electrostatic repulsion, Brownian motion, and others [55]. Thermodynamic stability of microemulsion means that these systems achieved minimum free energy and display no tendency to transform into separate phases. It is noteworthy that nanoemulsions are also considered as interesting and potentially applicable drug delivery systems and are a subject of numerous studies focused on efficient nose-to-brain drug transport. The current state of research on the application of these systems in nose-to-brain drug delivery is presented in other comprehensive reviews [36,57].

As it was mentioned, microemulsions are composed of polar and non-polar phases stabilized with an amphiphilic agent decreasing the interfacial tension between these two components. It is noteworthy that in the case of microemulsions the interfacial tension is extremely low. In order to obtain values that are close to zero, another agent enhancing the effects of surfactant is often needed. For this purpose, co-surfactants, low molecular weight compounds revealing good affinity to both phases, are applied. In pharmaceutical formulations, short-chain alcohols such as ethanol, isopropanol, and propylene glycol are common [58,59,60].

### 2.2. Formation Process and Microemulsion Stability

Another important feature of microemulsion is the spontaneous formation process, which does not require any significant amount of energy and is different from nanoemulsions prepared usually with the use of ultrasound or high-shear homogenization [61]. This phenomenon can be explained by thermodynamic properties of the system. The change in free energy associated with microemulsion formation can be described with the Gibbs–Helmholtz equation (Equation (1)),

(1) $$\Delta G_{form}=\Delta A\gamma_{o/w}-T\Delta S_{conf}$$

where $\Delta G_{form}$ is the change in free energy in the formation process, ΔA is the change in interfacial area between polar and non-polar phases, $\gamma_{o/w}$ is the interfacial tension, T is temperature, and $\Delta S_{conf}$ is the configurational entropy change [62]. In all spontaneous processes, $\Delta G_{form}$ must adopt negative values. As it was mentioned above, the interfacial tension is close to zero, which means that $\Delta A\gamma_{o/w}$ is also very low, even though interfacial area increases significantly because of formation of numerous small droplets. Taking into consideration the fact that droplet formation results also in an increase of entropy, it is obvious that $T\Delta S_{conf}$ > $\Delta A\gamma_{o/w}$. Therefore, $\Delta G_{form}$ in the case of microemulsions is negative. It is important to note that an extremely low interfacial tension is crucial for this process which explains why in most systems the presence of co-surfactant is necessary. This property is extremely important in terms of technological process and production costs as due to spontaneous formation no specific equipment requiring large amounts of energy is required.

It is noteworthy that spontaneous formation of microemulsions is related to the energy minimum they reach according to Gibbs-Helmholtz equation. As they exist in an energetically favorable state, microemulsions do not reveal the tendency to transform into some other system which is defined as thermodynamic stability. This is an important feature distinguishing microemulsions from other similar systems, including nanoemulsions or coarse emulsions. In the case of both latter ones, no thermodynamic stability is observed and both systems theoretically tend to transform into lower energy state. However, nanoemulsions display kinetic stability, which means that the transformation of nanoemulsion into energetically favorable state is very slow.

### 2.3. Classification of Microemulsions

Depending on the quantitative composition of the system, three different microemulsion types can be formed:

- Water-in-oil (W/O) with water as the dispersed phase and oil as the continuous one,
- Oil-in-water (O/W) with oil as the dispersed phase and water as the continuous one,
- Bicontinuous with water and oil forming interpenetrating three-dimensional domains without the possibility to discern internal and external phases.

It is noteworthy that bicontinuous systems are typical only for microemulsions, while W/O and O/W systems can be also observed in other dispersions, like coarse emulsions and nanoemulsions. This unique system is usually formed when polar and non-polar phases are present in similar amounts, while W/O and O/W systems are observed when higher amounts of oil and water are applied, respectively [63]. It is noteworthy that transformations between O/W and bicontinuous systems, as well as W/O and bicontinuous systems, may be observed as the result of an increase in water or oil content. Similar transformations can be achieved with temperature changes and are widely described as percolation transitions.

An alternative classification of microemulsions was proposed by Winsor [64]. According to this system, four microemulsions types can occur. Winsor I and II are O/W and W/O microemulsions, respectively. As explained above, they contain one phase dispersed in the form of droplets into another one. In pseudoternary phase diagrams commonly used to describe phase equilibria in microemulsions, Winsor I and II remain in equilibrium with water and oil phase, respectively. Winsor III is a middle phase bicontinuous microemulsion coexisting with both oil and water phases, while Winsor IV is a single phase microemulsion region occurring as a result of increase in surfactant content and it does not coexist with any other phase.

### 2.4. Applications

For many years, microemulsions have been investigated in numerous scientific and industrial areas. The properties of these systems, e.g., low viscosity, extremely low interfacial tension, small droplet diameter, and excellent solubilizing potential, can be utilized in many different ways. For example, because of small internal phase dimensions, they have been subjected in numerous studies as reaction media for nanoparticles synthesis. It is noteworthy that depending on the type of microemulsions and the selected reaction substrates and conditions, both inorganic [65,66,67] and polymer nanoparticles [68,69,70] can be obtained. In this approach, microemulsion droplets act as nanocontainers or nanotemplates for particle synthesis. It has been mentioned that low viscosity and Newtonian character of microemulsions, as well as their ability to dissolve various reaction substrates are useful properties for applying these systems as reaction media for nanoparticles synthesis [71]. Additionally, large interfacial area present in these systems can be advantageous for increasing the rate of catalytic processes, even though their complexity and possible interaction with reagents can be challenging [72].

Microemulsions are also employed in microemulsion electrokinetic chromatography (MEEKC), an analytical technique in which the analytes are separated based on the difference in polarity and electrophoretic mobility. In this method, O/W systems obtained with an anionic surfactant and n-butanol as co-surfactant are usually utilized. Depending on its polarity, the analyte is either localized in non-polar oil droplets or in the continuous phase, which reveals high polarity. Polar non-ionized analytes dissolved in an aqueous medium move towards the detector faster than the ones revealing lower polarity that are dissolved in oil droplets. Moreover, there are additional factors affecting the behavior of the analyzed substances. Cationic ones can interact with microemulsion droplets because of the negative charge localized on their surface while anionic solutes are repulsed by the droplets. It was shown that this method allows for quick and reliable separation of various chemical entities differing significantly in terms of polarity. Since the introduction of the technique in 1991 [73], it has been extensively investigated and modified [74,75].

Good oil solubilizing properties of microemulsions and their ultralow interfacial tension are utilized in the petroleum industry and the development of new cleaning products. In the petroleum industry, they are applied in enhanced oil recovery also known as tertiary oil recovery. In these methods, different substances are injected into oil reservoirs to increase the efficiency of the recovery process. In this case, microemulsions decrease capillary forces keeping oil droplets trapped inside rock crevices [76]. Cleaning applications include both household products and industrial cleaning processes [77]. Literature reports indicate that these systems can be considered as relatively inexpensive, safe, and effective cleaning media for challenging and delicate surfaces, like works of art [78].

One of the most extensively explored applications of microemulsions is in pharmaceutical technology. Numerous studies show that these systems can be effectively applied as drug carriers in topical and transdermal drug delivery, increasing drug absorption into the skin surface, and improving therapeutic efficacy [49,79,80,81,82,83,84]. Several theories presenting the possible explanation of this phenomenon have been presented [85]. It has been hypothesized that the increased efficiency of microemulsion-based therapeutics applied dermally can be related to increased solubilization capacity when compared to conventional dosage forms, as well as the specific composition of microemulsions. Surfactants and co-surfactants necessary for microemulsion formation can act as co-solvents for poorly water-soluble active ingredients and can also interact with the *stratum corneum*, the outermost barrier layer of the skin. It is noteworthy that numerous surfactants and low molecular weight alcohols frequently employed as co-surfactants in microemulsions are recognized as skin permeation enhancers, temporarily disrupting the organization of lipid molecules in the *stratum corneum* and facilitating the penetration of active ingredients through the skin [86,87,88,89]. Similar action can be exerted by surfactants and oil phase components [90]. Moreover, in the case of O/W microemulsions, drug-loaded oil droplets can act as a drug reservoir, thus maintaining a high concentration gradient between the applied formulation and the skin. The water phase is linked to the increased hydration of *stratum corneum,* which increases its permeability [85]. It was also shown that microemulsions can increase the bioavailability of drugs administered orally [91]. However, in oral drug delivery, self-microemulsifying systems (SMEDDS) are investigated more frequently. These systems are composed of oil, surfactants, and co-surfactants and form microemulsions in situ upon the contact with gastrointestinal fluid [92]. Few SMEDDS products, like Sandimmun Neoral^®^, have been successfully introduced to the pharmaceutical market [93]. In the case of cyclosporine, a poorly water-soluble drug, the application of a lipid-based carrier resulted in an increased bioavailability, improved dose-response linearity and reduced food effects as well as inter- and intra-individual variability reduction [94]. Microemulsions also seem to be interesting carriers in parenteral and ophthalmic formulations, increasing solubility of hydrophobic drugs and improving bioavailability. Moreover, they form spontaneously and are transparent, which is particularly important in formulations administered to the conjunctival sac. However, in both of the administration routes, the selection of available excipients is limited and the design of microemulsions meeting the standards of parenteral and ophthalmic products can be challenging. For example, surfactants like Kolliphor^®^ EL (previously marketed as Cremophor^®^ EL, BASF) or polyoxyethylene alkyl ethers may cause serious side effects after parenteral administration including anaphylactic reactions or hemolysis [95]. It is also important to note that high amounts of surface-active agents in ocular products can cause toxic effects. Co-surfactant type and concentration in microemulsion considered for potential parenteral and ophthalmic administration should be also carefully chosen as some of them in higher amounts may cause pain upon injection or hemolysis and are not well tolerated by the eye [96]. However, despite the described difficulties, ocular and parenteral microemulsions are still extensively investigated and employed to obtain innovative drug delivery systems, like microemulsion-laden contact lenses [97,98,99].

## 3. Nasal Cavity as Drug Administration Site

### 3.1. Anatomy and Physiology of Nasal Cavity

The nasal cavity, pharynx, and larynx together form the upper respiratory tract. The nasal cavity consists of two irregular spaces separated from each other by the septum and confined by different bones joined with connective tissue. The upper parts of both chambers open to nostrils while the lower parts connect to the nasopharynx which is the upper part of the pharynx [100]. The volume of each chamber is approximately 13 mL, while the surface area is about 150 cm^2^ [101]. Both cavities consist of three parts including the vestibule, the olfactory region, and the respiratory region which is the largest of them. The nasal vestibule, the external most part connecting to the nostrils, is the smallest of the mentioned regions. Its area is approximately 0.6 cm^2^. Inside the respiratory part on the outer walls, the superior, middle, and inferior turbinates are located. Upon the contact with inhaled air they introduce turbulent flow, which improves its contact with nasal mucosa. It is also noteworthy that this part of the nasal cavity is connected to four sinuses. Located above the turbinates is the olfactory region, which is separated from the brain by a horizontal perforated bone plate known as cribriform plate. In its orifices, nerves coming from olfactory bulb are located. Cribriform plate is considered as the only area in the body allowing direct contact of the central nervous system with an external mucous membrane [100].

It is estimated that about two-thirds of the surface of the vestibules is covered with skin, while their internal part is lined with squamous epithelium and transitional epithelium. In the remaining parts of nasal cavity, two types of epithelium are present. The thinner tissue known as the olfactory epithelium can be only found in the upper chamber recognized as the olfactory region. The cilia present in this type of epithelium are longer and do not move, which is related to much slower mucus turnover [7]. The olfactory epithelium contains chemoreceptive olfactory neurons with their supporting cells, known as sustentacular cells, and basal cells. Sustentacular cells are columnar cells equipped with microvilli. They exhibit mechanical and metabolic function and also regulate the ionic equilibrium in the mucus. It is noteworthy that they reveal high cytochrome P-450 activity, which is related to quick metabolism of the inhaled drugs and other substances [102]. Basal cells are located in the epithelial basement membrane and also act as mechanical support for other types of cells. They can be transformed into the other types of cells present in the olfactory epithelium. The olfactory neurons are the most important component of the olfactory epithelium because of their role in reception and transformation of chemical olfactory signals into neural ones. They are bipolar, ciliated cells containing chemoreceptors responsible for the olfaction process. Their axons are gathered in bundles and cross the orifices in the cribriform plate, while their dendrites are located in the olfactory bulb [103]. The diameter of the olfactory neuronal axons ranges from about 100–700 nm [104], which theoretically enables nanoparticle transport [105]. According to another hypothesis, active ingredients can be transported through the vessels running along the axons bundles [101]. Therefore, this pathway is considered as an option to deliver active pharmaceutical ingredients from nasal epithelium to the olfactory cortex.

The main tissue lining about 80–90% of the surface area in the nasal cavity [102] is the respiratory epithelium. It contains basal cells and columnar cells either non-ciliated or equipped with cilia, structures similar to hair responsible for the mucus transport towards the pharynx. Among them, goblet cells secreting mucus are present. It should be noted that the epithelial cells present in the nasal cavity are covered with microvilli, which significantly increase the surface area available for drug absorption. High contact area between air and the mucous membrane is also important for efficient air filtration, as well as for increasing its humidity and temperature before reaching the lower respiratory tract. On the other hand, high surface area and dense vascular network typical for respiratory mucosa make this region suitable for systemic drug absorption [102].

Mucus produced by goblet cells contains water, mucin, salt, and a mixture of other proteins including albumin, lactoferrin, immunoglobulins, lysozyme, and a small portion of lipids [101]. It is organized in two layers. The upper viscous part is transferred by organized cilia movements to the nasopharynx. The mucus layer acts as a protective barrier for lower respiratory tract, preventing penetration of pathogens and particles to lower parts of respiratory system. Solid particles exceeding 3–10 μm diameter stick to the viscous gel and are moved to the nasopharynx [101,106]. Mucin, the main gel-forming component of mucus, is a glycoprotein containing the ability to form disulfide bonds, which is the cause of viscosity increase. Due to alternating hydrophilic and hydrophobic domains present in the molecule, mucin can effectively bond both hydrophilic and hydrophobic compounds delivered to the nasal cavity, which might affect their permeation through mucous membrane. Alternatively, mucoadhesion forces between mucous membrane and the applied formulation can prolong residence time at the administration site and increase bioavailability of active ingredient [107].

### 3.2. Drug Delivery Pathways

The nasal cavity can be employed as a drug administration site for the active ingredients exerting local effects, e.g., corticosteroids and vasoconstricting or antihistamine agents commonly applied in allergic or infectious conditions. However, high surface area due to the presence of microvillii in nasal epithelium, as well as its strong vascularization, are advantageous features in terms of systemic drug delivery [108]. Among currently marketed intranasal products exerting systemic action, 5-HT receptor agonists applied in migraine, sedative, and hypnotic agents applied to treat insomnia, opioids, peptides like desmopressin or calcitonin, testosterone, and many others can be listed. It must be emphasized that drug absorption rate from nasal cavity is crucial for the therapeutic efficacy. One of the most important factors affecting drug permeation through nasal mucosa is active ingredient polarity. It was shown that permeation is usually faster for lipophilic molecules, which leads to similar pharmacokinetic profile as in the case of intravenous injection [109]. The described effect was observed for fentanyl [110,111] and midazolam [112,113]. The opposite mechanism was shown for polar drugs [114]. Another factor limiting the permeation rate is drug molecular weight. It was shown that the bigger the drug molecule, the slower the absorption from the nasal mucosa. For example, large peptide molecules usually do not exceed bioavailability of 1% after nasal administration [109]. Additional effects might be observed due to the rapid clearance mechanism associated with nasal mucosa physiology. If the drug deposited on the nasal mucosa is not absorbed instantly, it will be transferred to the nasopharynx. Therefore, the composition and properties of the carrier may be crucial for the therapeutic effect achieved after intranasal drug administration.

Considering the delivery of active pharmaceutical ingredients to brain, a few different pathways should be mentioned on the basis of absorption rates. It must be understood that both olfactory and respiratory areas in the nasal cavity are highly vascularized. The olfactory region is supplied with blood by the branches of the ophthalmic artery, while the respiratory region by the branches of the maxillar artery. Therefore, the drug deposited on the mucous membrane can be also absorbed into the bloodstream and reach the brain and the other organs via systemic circulation [115]. Another option is the absorption of the drug into the venous circulation and quick transport to the carotid artery. In this way, the active ingredient can be delivered to the brain and the spinal cord by the process known as counter-current transfer [115,116]. However, considering systemic circulatory-mediated drug transport, it should be kept in mind that the BBB must be crossed in order to reach the brain tissue. As previously mentioned, effective drug delivery through this barrier is challenging and for most of the active ingredients, particularly macromolecular ones, practically impossible. Therefore, numerous studies and reviews focus on the alternative routes, including also neuronal pathways involving nasal cavity. However, it is of importance to remember that the administered active ingredient can be delivered via more than one pathway. The prevalence of one over the other can depend on different factors including physicochemical properties of the drug, the composition of the carrier, and the application method [102,117].

#### 3.2.1. Olfactory Pathway

As highlighted earlier, the olfactory region in the nasal cavity is the only area in the body providing relatively close contact of the central nervous system with the external mucosa. Therefore, it has been extensively investigated as an attractive option for the delivery of active ingredients directly to the brain. Among possible advantages of this approach there are ease of administration, quick onset of action, and reduced systemic effects. Considering the exact mechanism of this phenomenon, three different pathways across the olfactory epithelium have been described. In the transcellular pathway, the drug crosses epithelial cells, particularly sustentacular cells by endocytosis or passive diffusion. It is important to note that the latter mechanism is favorable for lipophilic actives and the rate of this process strongly depends on the polarity of the drug. Extracellular transport involves the tight junctions between the sustentacular cells or spaces between the olfactory neurons and sustentacular cells. The olfactory neurons undergo frequent regeneration, which leads to the weakening of the nasal barrier and increase in its permeability [118]. In the described process, the drug diffuses through water channels without crossing the lipophilic cell membranes. Therefore, this mechanism is favorable for the hydrophilic actives exhibiting low molecular weight. The third possible pathway involves the transport through olfactory neurons with absorption of the drug into the cell by endocytosis or pinocytosis [101]. This route is considered as kinetically slow and inefficient in terms of drug delivery to olfactory bulb. Moreover, drug internalization can be considered as potentially harmful for the olfactory neurons, which may lead to toxic effects and impaired olfactory functions [119].

It is noteworthy that apart from passive transport mechanisms, some active influx or efflux transporters may also be involved in drug delivery to olfactory bulb with the former promoting the uptake of active ingredients, while the latter ones displaying barrier functions are responsible for the removal of exogenous substances [119].

#### 3.2.2. Trigeminal Nerve Pathway

Another possible approach to drug delivery to brain involves transport through trigeminal nerve, the fifth and the largest cranial nerve responsible for gathering sensory signals and partially for motor functions in facial area. Its endings are present in the respiratory and olfactory regions of the nasal cavity and part of it leads to central nervous system that may be useful as a delivery route. It enters the brain via two different routes, through the cribriform plate and through a foramen located at the pons level [101,103]. According to the literature, in this case the transport may occur either by transcellular or extracellular paths [120,121].

**Figure 2:**
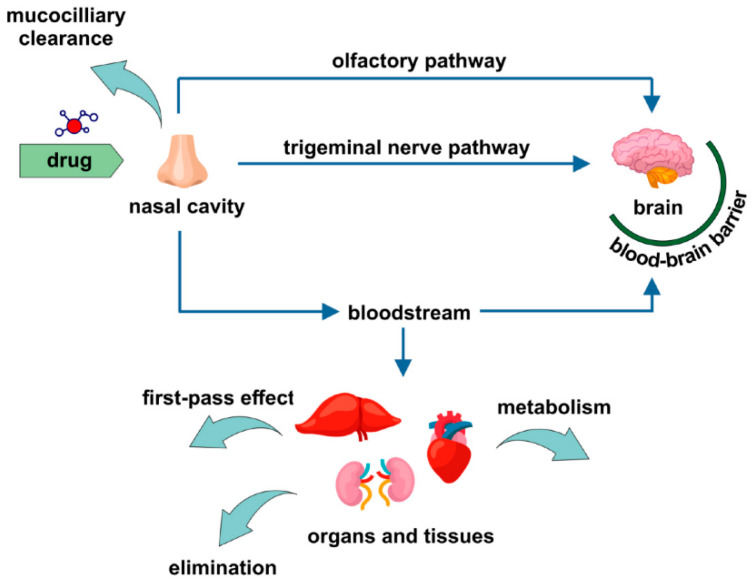
Drug delivery pathways related to intranasal administration. Reproduced with permission from [101,102], Taylor and Francis Group, 2013 and Elsevier, 2018, respectively.

Possible drug delivery pathways following the intranasal administration are depicted in Figure 2.

## 4. Transnasal Formulations in Brain Targeting

The formulation selected for intranasal administration can have a significantly effect on the efficacy of the administered drug. Nasal cavity seems to be an attractive site for drug administration, exhibiting a high surface area and a dense vascular system capable of transporting active ingredients through neuronal pathways important for the effectiveness and rapid onset of action. Drugs administered nasally can avoid the hepatic first-pass effect, which leads to higher bioavailability. Moreover, nasal formulations are generally well accepted and considered as convenient by patients. However, this delivery route reveals also some important disadvantages limiting the efficacy of nasal formulation, like rapid removal of the substances deposited on nasal mucous membrane, enzymatic degradation of the active ingredient, toxic effects related to irritation of the nasal mucosa, and insufficient permeability [101]. Therefore, the design of the delivery system is crucial for safety and effectiveness of the therapy. As this delivery route has been extensively investigated in the latest research, numerous types of formulations have been analyzed for potential intranasal drug delivery systems. The literature reports polymer [122] and lipid nanoparticles [123], micelles [39], nanoemulsions [124], liposomes [125], and many other drug delivery systems in addition to mucoadhesive formulations [126] and in situ gelling systems [38]. The presence of mucoadhesive excipients in the product could cause extended residence times at the administration site, which is particularly important in the case of nasal mucous membrane revealing high mucociliary clearance that can easily remove actives from the nasal cavity and lead to reduced absorption and decreased therapeutic efficacy. In situ forming gels contain stimuli-sensitive excipients reacting to the conditions in the nasal cavity resulting in an increased formulation viscosity. In this approach, the formulation is administered in a liquid state that allows for good distribution in the nasal cavity, is transformed into a gel that extends its contact with nasal mucous membrane. These approaches are combined with encapsulation of active ingredients in the nanoparticulate carriers mentioned above [127,128].

The described approaches requiring brain targeting usually involve therapeutic agents useful in the treatment of various neurological and psychiatric disorders, including chronic neurodegenerative disorders, like Alzheimer’s and Parkinson’s diseases, epilepsy, schizophrenia, and many others. According to the World Health Organization (WHO), neurological disorders affect numerous people in the world. It is estimated that about 50 million people suffer from different kinds of dementia and 60–70% of this population are Alzheimer’s disease sufferers. The number of dementia cases is expected to reach 150 million by 2050 [129]. The second most common neurodegenerative condition is Parkinson’s disease. Currently, it is considered as a fastest growing neurological disorder and its prevalence is expected to reach approximately 13–14 million cases by 2040 [130]. According to WHO data, approximately 50 and 20 million people worldwide suffer from epilepsy and schizophrenia, respectively [131,132]. In all of the mentioned disorders, effective and safe treatment options are very important. Taking into consideration the fact that nasal drug administration is convenient for the patient and the administered drugs can bypass the blood–brain barrier and quickly reach the targeted site, nose-to-brain formulations appear to be attractive delivery systems. However, the nasal cavity also exhibits some drawbacks as a potential administration site and the most important challenge limiting the drug efficacy is due to poor absorption from the nasal mucous membrane. Therefore, numerous studies focus on the design of novel drug carriers, including microemulsions, enabling an efficient drug transport across nasal mucosa.

### 4.1. Neurodegenerative Disorders

Alzheimer’s disease (AD) is a neurodegenerative disorder resulting in symptoms of dementia, including impairment of cognitive functions and behavioral changes disturbing daily activities. The severity of the observed symptoms gradually increases, eventually leading to premature death [133]. It is estimated that only about 5–10% of AD cases are related to hereditary factors, while the other ones are most probably caused by the combination of genetic, environmental, and lifestyle factors [134]. Several hypotheses describing the pathogenesis of the disorder have been formulated; however, the exact causes are still not known. One of the most important neurological symptoms of AD is the formation of amyloid plaques in the brain tissue. It is hypothesized that this occurs as a result of an altered amyloid precursor protein cascade leading to formation of a highly fibrillogenic product. Another possible mechanism of neurodegeneration is related to neuronal tau proteins, which are highly phosphorylated in the AD-affected brain. As a result, neurofibrillary tangles hypothetically related to neurodegeneration are observed. According to the glutamate theory, *N*-methyl-d-aspartate (NMDA) receptors localized in AD are hyperactive leading to enhanced binding of glutamate and glycine, disturbances in ionic equilibrium, and eventually to cell death. Moreover, in AD, some abnormalities in acetylcholine levels in brain were observed, which can contribute to the cognitive functions impairment [134]. In Parkinson’s disease (PD), a progressive loss of dopaminergic neurons in *substantia nigra* in the central nervous system associated with dopamine level depletion are observed. As a result, progressive impairment of motor functions accompanied with tremors at rest and muscle rigidity occur. Moreover, significant psychiatric symptoms, like dementia, cognitive functions impairment, anxiety, and depression are observed [133]. So far, most of the drugs most commonly applied in AD and PD are administered orally, even though it is associated with side effects and limited permeation through the blood–brain barrier, the only exception being transdermal patch containing rivastigmine [135]. Nanotechnology-related studies focusing on the design of novel drug delivery systems for intranasal administration involve microemulsion-based media among other colloidal systems.

Several studies on nose-to-brain delivery of rivastigmine, a reversible and non-competitive acetylcholinesterase inhibitor used for enhancing cholinergic neurotransmission, have been recently presented according to its application in mild to moderate AD and PD-related dementia. The main drawbacks of the drug are its low oral bioavailability, degradation in gastrointestinal tract and first pass metabolism. Shah et al. [136] investigated nasal mucoadhesive microemulsions (ME) for nose-to-brain delivery of rivastigmine hydrogen tartrate (RHT). The analyzed systems were composed of Capmul^®^ MCM EP (oil), Labrasol^®^ (surfactant), Transcutol^®^ P (co-surfactant), and water. In order to overcome nasal mucociliary clearance and to extend residence time, two types of mucoadhesive components, namely chitosan (CH-ME) and cetyltrimethylammonium bromide (CTAB-ME), were used. The ex vivo diffusion studies were performed with Franz diffusion cells equipped with excised goat nasal mucosa. Aqueous drug solution (DS) was used as a reference. The results of the 8h experiment showed the order of diffusion coefficient as follows CH-ME > CTAB-ME > ME > DS, which clearly depicted the prevalence of chitosan-based microemulsion over the other formulations. The authors state that interactions of chitosan with mucosal tight junctions can be one of the explanations for diffusion improvement. Additionally, the nasal cilio-toxicity test showed no evidence of harmful influence of the formulations on the mucosa.

On the basis of the previous studies, Shah et al. [42] developed nasal spray with RHT. In vivo pharmacokinetic experiments on Sprague Dawley rats revealed that RHT concentration in brain following intranasal administration of CH-ME was found to be higher at all the time points compared to ME and DS. The pharmacokinetic results were in agreement with qualitative biodistribution assay by means of gamma scintigraphy visualization.

In a subsequent study presented by Khunt et al. [137], RHT nasal bioavailability was investigated after its administration in the form of microemulsions enriched with butter oil (BO) and fish oil (FO). The concentration profiles of RHT in rat brains showed the potential for penetration enhancement of BO and FO, with slight prevalence of fish oil. It was also observed that after nasal application of RHT microemulsion, the drug reached higher concentration than after intravenous injection. Additionally, the authors performed in vitro test to evaluate the protective role of MEs against amyloid-β (1–42) oligomer induced toxicity in IMR 32 cell line. Unfortunately, there was no significant increase in cell viability compared to the effect of pure drug.

As a continuation of the previous research, Katdare et al. [138] prepared and evaluated similar formulations with galantamine hydrochloride, a reversible and competitive cholinesterase inhibitor. The microemulsion was composed of Capmul^®^ MCM EP (oil), Tween^®^ 80 (surfactant), Transcutol^®^ P (co-surfactant), and water. The authors performed several cell-based anti-oxidative stress assays, namely glutathione assay, nitrite assay, and lipid peroxidation assay, to check the protective effects of the developed formulations. Both the cell viability test and in vivo animal brain delivery studies showed the efficacy of formulations in the order of ME < BO-ME < FO-ME. Addition of FO showed clearly higher impact on brain delivery when administered by intranasal route than intravenously. The study confirmed the potential benefits of FO and BO co-administration not only for enhancing the permeation of drugs across the blood–brain barrier, but also for decreasing the oxidative stress in cells.

In another study, Khunt et al. [139] investigated the influence of omega-3 fatty acids (O3FO) and butter oil (BO) on nasal delivery of donepezil hydrochloride, a non-competitive cholinesterase inhibitor. The authors observed higher percentage of nasal diffusion for microemulsion enriched with BO (71.22%) and O3FO (62.16%) in comparison to simple ME (59.69%) and drug solution (55.01%). In vitro cell permeability study confirmed the advantage of BO and O3FO. In the case of brain bioavailability after intranasal administration investigated with the use of a Sprague Dawley rat model, O3FO turned out to be more effective than BO.

Jogani et al. [140] developed mucoadhesive microemulsion-based system for brain delivery of tacrine, a reversible cholinesterase inhibitor used in the treatment of AD. The microemulsion (TME) was obtained with the use of Labrafil^®^ M 1944 CS (15%), Cremophor RH 40 (41.25%), Transcutol^®^ P (13.75%), and water (30%). For preparation of mucoadhesive microemulsion (TMME) Carbopol^®^ 934 P was used. For visualization of drug distribution in BALB/c mice, the formulation was radiolabelled using ^99m^Tc. Tacrine biodistribution was investigated after administration of the solution intravenously and intranasally in comparison to intranasal microemulsion and intranasal mucoadhesive microemulsion. The obtained results clearly indicated that the latter one exhibited the most promising properties in terms of possible application and direct drug delivery to the central nervous system. Additionally, to evaluate the influence of the formulations on learning and memory capacities the authors performed the Morris water maze for scopolamine-induced amnesia model in mice. It turned out that the fastest recovery (three days) was observed for TMME, while for TME and tacrine solution the time was longer (four days). The worst results were obtained for formulations administered intravenously (no recovery after four days).

Some studies were recently performed for intranasal delivery of donepezil hydrochloride. Espinoza et al. [141] developed a microemulsion using castor oil, Labrasol^®^, Transcutol^®^ P, and propylene glycol. The ex vivo studies with the use of porcine nasal mucosa showed that after 6 h of treatment, more than 32% of the drug retained in porcine nasal mucosa. The authors assumed that nasal mucosa could act as a reservoir for drugs, which can be useful when sustained release is desired.

The studies available in the literature also involve active ingredients which can be potentially useful in AD therapy. Chen et al. [142] prepared a pH and thermosensitive gel, based on microemulsion for nasal delivery of Huperzine A (hup A), a reversible acetylcholine esterase inhibitor and NMDA receptor agonist extracted from Chinese plant *Huperzia serrata*. The gel base consisted of Pluronic^®^ F127. Accounting for its rapid erosion upon contact with body fluids, two viscosity-enhancing polymers were added, namely Pluronic^®^ F68 and chitosan. The latter acted also as a mucoadhesive and pH-regulatory component. The microemulsion composed of 1,2-propanediol, castor oil and Cremophor RH40. The pharmacokinetic study in vivo was evaluated by microdialysis with the use of Sprague-Dawley rats. The authors tested four types of formulations: Solution of hup A, hup A microemulsion, hup A microemulsion temperature-responsive in situ gel, and hup A microemulsion temperature/pH dual-responsive in situ gel. The solution of hup A was used as reference and was administered intravenously, while the other formulations were applied intranasally. After nasal administration, both the plasma and brain concentration profiles showed the evidence of sustained and prolonged release when compared to the solution, however they reached lower concentrations. It also turned out that the gels showed prevalence over the microemulsions by increasing absolute nasal bioavailability of hup A. The best results were obtained for the gel containing chitosan, which was attributed to its ability to enhance the permeability of the membrane structure.

Another substance potentially useful in AD treatment is morin hydrate. It was shown that it can inhibit the deposition of amyloid-β in the brain, as well as reduce the abnormalities related to tau protein phosphorylation leading to the formation of neurofibrillary tangles. Moreover, it exhibits some antioxidant activity. Sharma et al. [143] developed the micremulsion for murine nasal administration. The ME was composed of Capmul^®^ MCM, Cremophor EL, PEG-400, and water. After intranasal administration, brain and blood drug concentrations were higher for morin loaded microemulsion than those observed for drug solution. Additionally, a significant increase in memory of wistar rats was noticed with streptozotocin-induced dementia after 21-day treatment.

**Figure 3:**
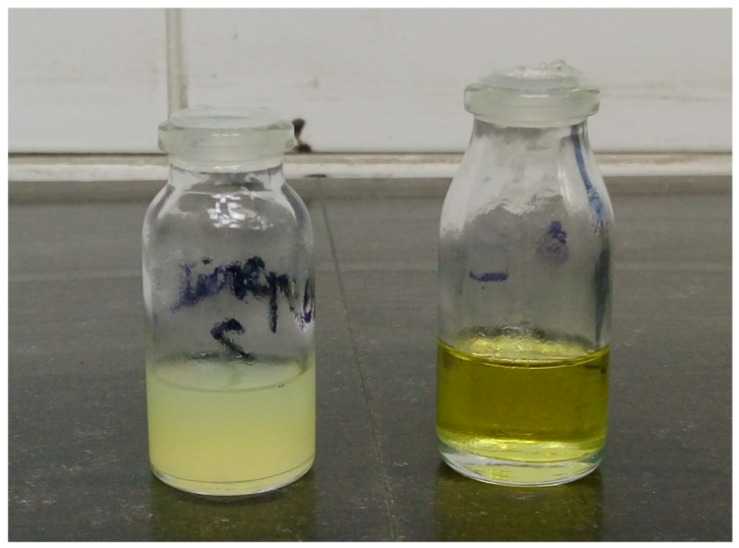
Photograph of the microemulsion formulation (F1) on the right and the composite formulation (F6) to the left. Reprinted with permission from [144], Elsevier, 2016.

Nasr and Wabdan [144] prepared comprehensive studies for nose-to-brain delivery of two cognitive enhancers, vinpocetine and piracetam. The authors developed various types of formulations including microemulsion, liposomes, ethosomes, transfersomes, and transethosomes. The microemulsion was composed of Tween^®^ 20, oleic acid, ethanol, and water. Additionally, the microemulsion/vesicular system was prepared with the use of soybean lecithin—Epikuron™ 200 (Figure 3). The ex vivo experiments on sheep nasal mucosa has shown that the amount of released piracetam was two-fold higher than vinpocetine, which was attributed to the fact that the first one was present in the external phase of the formulations. Y maze test and passive avoidance test on male rats were conducted in order to confirm beneficial effect of the formulations on the cognitive functions. In both cases, the nanoformulations showed significant improvement.

Mandal et al. [145] investigated intranasal mucoadhesive microemulsion with ibuprofen as neuroprotective agent in Parkinson’s disease. Capmul^®^ MCM, Accenon^®^ CC, and Transcutol^®^ were selected as oil, surfactant, and co-surfactant, respectively. To achieve a mucoadhesive effect, polycarbophil was applied. The quantitative composition of the analyzed system was optimized with the use of response surface methodology with globule size, viscosity, permeation flux, and lag time in ex vivo permeation studies as response parameters. The optimized formulation was used for in vivo studies performed with the use of mice model. In the tests, nasal ciliotoxicity and neuroprotection in 1-methyl-4-phenyl-1,2,3,6-tetrahydropyridine (MPTP) model of Parkinson’s disease were evaluated. MPTP was applied as a toxic agent reducing the level of striatal dopamine as it is observed in the neurodegenerative disorder. It was found that the analyzed system did not reveal toxic properties and was suitable for nasal administration. Striatal dopamine levels reduced by the toxic agent were increased after nasal administration of ibuprofen-loaded microemulsion system. It was also found that microemulsion-treated animals revealed better muscular coordination compared to the group without ibuprofen-loaded formulation. The comparison between the microemulsion with and without the mucoadhesive agent showed better muscular coordination in the case of formulation without polycarbophil. However, in the animal study, the groups treated with ibuprofen-loaded microemulsions with or without polycarbophil were compared only to intoxicated and non-treated group and non-intoxicated and non-treated group. Considering the fact that no drug-loaded formulation without microemulsion was used in the study, it is difficult to evaluate the significance of microemulsion carrier in nose-to-brain drug delivery, even though the potential neuroprotective activity of the analyzed formulations was proven.

The studies describing the application of microemulsion-based media in nose-to-brain delivery of therapeutic agents useful in the management of neurodegenerative disorders are summarized in Table 1.

### 4.2. Epilepsy

Epilepsy is a chronic neurological disorder characterized by the occurrence of partial or generalized seizures manifesting with loss or disturbances of consciousness with or without convulsions [146,147,148]. The seizures are a consequence of abnormalities related to the electrical activity of brain neurons. The described effect is also associated with abnormal levels of neurotransmitters, including an increase of glutamate and a decrease of gamma-aminobutyric acid (GABA) [148]. Antiepileptic drugs are used to reduce the frequency and severity of the seizures but they do not remove the causes that are still not completely understood [149]. It is worth noting that these drugs usually cause severe cerebral and systemic side effects resulting from relatively high doses needed to cross the blood–brain barrier. Another challenge encountered in epilepsy management is the necessity to strictly monitor and individualize the dosing scheme. Additionally, even combined antiepileptic therapy with more than one drug may remain only partially effective. It is estimated that one third of the patients suffering from epilepsy still experience seizures [147]. Prolonging seizures can be dangerous and require urgent pharmacological intervention. For this purpose, rectal or nasal medications can be applied [150]. In such a condition, rapid onset of action is crucial for the prevention of the spread of the electrical discharge and the complications associated with prolonging epileptic seizure. Intranasal formulations are an attractive treatment option, as they provide quick drug absorption from nasal mucosa and direct transport to the brain [147].

Benzodiazepines are important therapeutic agents introduced to the pharmaceutical market in 1960s [151]. They are considered relatively safe and rapid-acting therapeutics particularly useful in seizure-related emergencies. However, the use of benzodiazepines is associated with several drawbacks, including sedation, cognitive functions impairment, interactions with other drugs, and tolerance development with possible addiction following. They interact with GABA receptors enhancing inhibitory action of GABA neurotransmitter [151].

Florence et al. [146] published the results of a study aiming at the formulation of an intranasal, mucoadhesive oil-water (O/W) microemulsion for nose-to-brain delivery of clobazam, an anticonvulsant benzodiazepine drug used for the treatment of different epilepsy types and applied in some psychiatric disorders manifesting with anxiety. The obtained microemulsion contained Capmul^®^ MCM as the oil phase and the combination of polyoxyethylene-6-caprylic and capric glycerides (Acconan^®^ CC6) and Tween^®^ 20 as the surfactant-cosurfactant mixture. In order to obtain mucoadhesive properties, Carbopol^®^ 940P was added. An in vitro study performed with excised sheep mucosa revealed the highest permeation coefficient for clobazam-loaded microemulsion with mucoadhesive agent, followed by non-mucoadhesive microemulsion. Both formulations performed better in terms of drug permeation than the plain solution used as reference. The beneficial effects of mucoadhesive polymer were ascribed to the possible interaction with tight junctions in mucous membrane. In vivo studies performed with radiolabeled samples also indicated the highest brain/blood ratio at all time points for the polymer-enriched formulation. The experiment performed with pentylenetetrazole-induced seizures in mice revealed that non-mucoadhesive microemulsion had similar efficacy as an intravenous injection, while polymer-loaded microemulsion produced significantly prolonged effect and provided prolonged protection from convulsion-inducing agent. The described systems can be considered as an alternative for intravenous formulations used as an emergency treatment in seizures. It should be also emphasized that the proposed approach could potentially allow reducing the dose or dosing frequency of the drug.

Another benzodiazepine applied in epilepsy management is lorazepam, usually administered intravenously and approved by US Food and Drug Administration (FDA) for the treatment of status epilepticus [151]. It exhibits low solubility in water and extensive hepatic metabolism known as the first-pass effect. Moreover, during intravenous administration precipitation of the drug may be observed, which causes pain at the injection site. In the study presented by Shah et al. [152], microemulsion with lorazepam was designed. Carbopol^®^ and gellan gum were added to the formulation to obtain in situ gelling system and prevent rapid removal of microemulsion from the administration site. As an oil phase Capmul^®^ MCM was used and as surfactant and co-surfactant Nikkol™ PBC-34 and Transcutol^®^ P were applied, respectively. In ex vivo permeation studies performed with excised goat nasal mucosa, it was revealed that the permeation rate was the highest in the case of microemulsion without polymers, while the lowest one was recorder for plain drug solution, which was related to poor lorazepam solubility. The described formulations were also used in pharmacodynamic activity studies including behavioral tests. It was shown that the investigated formulations acted faster and longer as an anxiolytic agent than marketed formulation used as a reference. Moreover, in situ gelling formulation extended the duration of sleep after lorazepam administration and provided quick onset of action. It was concluded that the addition of gelling polymers reduced mucociliary clearance leading to rapid removal of the drug administered to the nasal cavity.

Ramreddy et al. [153] prepared mucoadhesive microemulsions for nasal delivery of diazepam, the benzodiazepine agent most commonly applied in seizure emergencies. The investigated mucoadhesive microemulsions were composed of oleic acid, Tween^®^ 80, propylene glycol, water, and chitosan. In the studies conducted with the use of an animal model, the efficacy of microemulsion-based formulations was evaluated and compared to the performance of marketed intravenous product and plain drug solution administered intranasally. The brain/blood ratios obtained for microemulsion and polymer-enriched microemulsion were higher compared to the reference systems, which indicated beneficial effects related to the application of microemulsion as a drug carrier. It was also shown that the addition of mucoadhesive component can significantly increase the efficacy of intranasal drug delivery system.

Another therapeutic agent applied both in epilepsy and psychiatric disorders is carbamazepine. It is commonly administered orally, which is associated with side effects including gastrointestinal disturbances, dermatological reactions, liver and respiratory problems, etc. [154]. Another challenge related to oral administration of the drug is its poor solubility in water and poor absorption from gastrointestinal tract.

Intranasal formulations with carbamazepine designed for direct brain targeting were investigated by few scientific groups. All of the presented studies were based on the hypothesis that microemulsion-based intranasal delivery could result in direct and rapid nose-to-brain transport of carbamazepine in comparison to available oral formulation and parenteral carbamazepine solution. In this way, the therapeutic effect of the drug could be improved with possible reduction of side effects and therapy costs.

**Figure 4:**
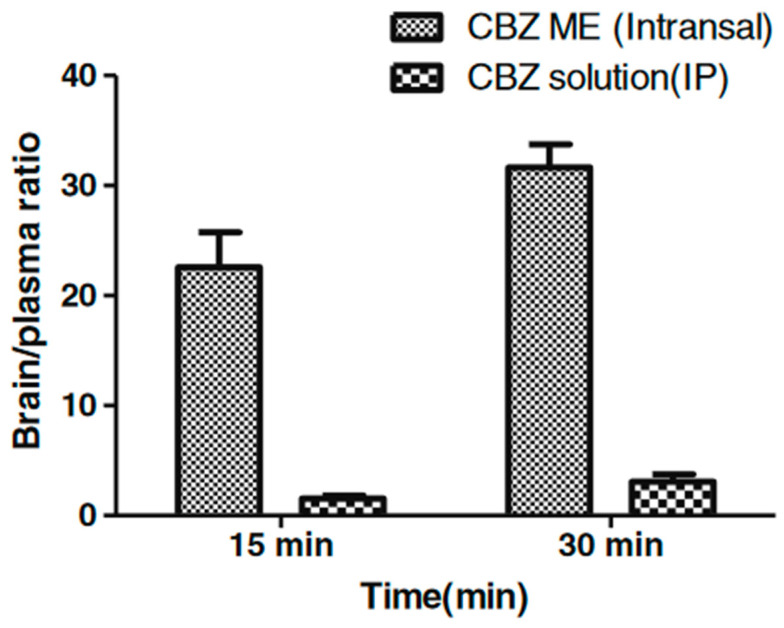
Brain/plasma ratios for carbamazepine-loaded microemulsion (CBZ-ME) (intranasal) and intraperitoneal carbamazepine solution (CBZ solution intraperitoneal IP). Reprinted with permission from [154], Springer, 2013.

Acharya et al. prepared and evaluated intranasal oil in water microemulsions with oleic acid as oil phase, Tween^®^ 80 as surfactant, and propylene glycol [155] or Transcutol^®^ [154] as co-surfactant. An ex vivo permeation study performed with the use of excised sheep mucosa revealed faster diffusion of carbamazepine incorporated in both microemulsion carriers compared to plain carbamazepine solution prepared with mixture of polyethylene glycol 400 (PEG400) and water. In the first step, the permeation was faster, which was most probably related to the presence of solubilized drug in the external phase of microemulsion. In the further stages of the experiment, the drug permeation was slower, which was ascribed to the release of the drug from microemulsion droplets. Ciliotoxicity studies performed with excised sheep mucosa revealed no negative effects upon the contact with the investigated formulations. In vivo investigations conducted with mice subjected to electrical shock in order to induce convulsions revealed that the analyzed formulations reduced the intensity of seizures and reduced the recovery time after the seizure. In both cases, the obtained results were similar to those observed after intraperitoneal administration of the drug solution. However, the analysis of brain/plasma ratio performed for the system with Transcutol^®^ revealed much higher carbamazepine concentrations in brain tissue after intranasal microemulsion administration compared to plain solution administered intraperitoneally. The obtained results (Figure 4) indicate the presence of alternative nose-brain pathway allowing for rapid and selective drug uptake.

Patel et al. [156] presented the study focused on carbamazepine-loaded microemulsion containing Labrafil^®^ M1944 as an oil phase and mixture of Cremophor^®^ RH 40: Transcutol^®^ P (4:1) as surfactant-cosurfactant system. The selected microemulsion was enriched with mucoadhesive (polycarbophil). The obtained systems were subjected to ex vivo drug permeation study performed with excised sheep mucosa, ciliotoxicity test, and ex vivo mucoadhesion studies. The obtained results indicated low irritancy of the investigated systems and enhancement of mucoadhesive properties in the case of polycarbophil-loaded formulation. However, an ex vivo permeation study revealed no statistically significant differences between the investigated systems and carbamazepine solution.

**Figure 5:**
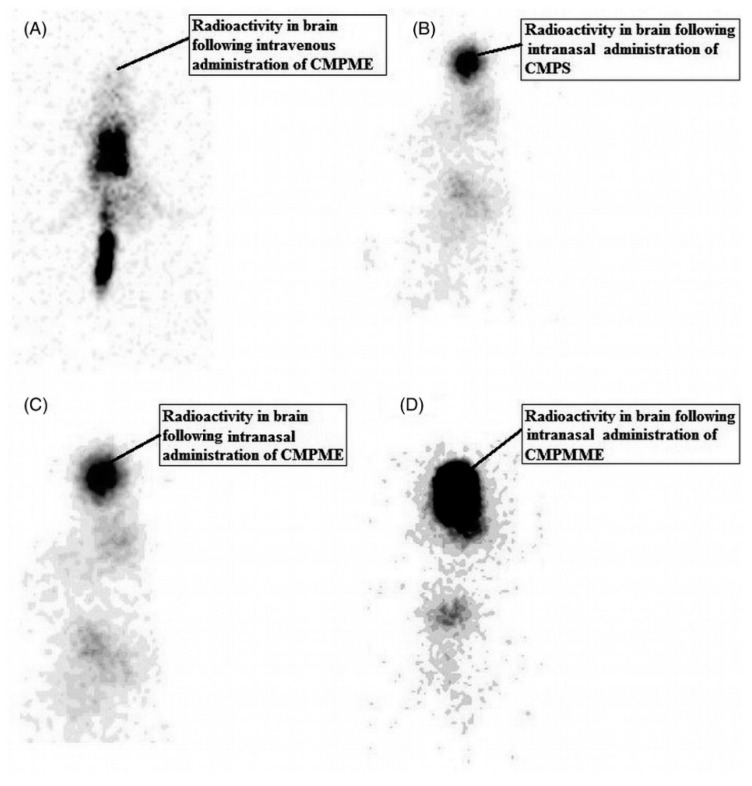
Gamma scintigraphy of antero-posterior (AP) view of rat following intravenous administration of radiolabeled carbamazepine-loaded microemulsion (**A**), intranasal administration of radiolabeled carbamazepine solution (**B**), radiolabeled carbamazepine-loaded microemulsion (**C**), and radiolabeled carbamazepine-loaded mucoadhesive microemulsion (**D**). Reproduced with permission from [157], Taylor and Francis, 2013.

In another study [157], the same research group investigated the same microemulsion using an in vivo animal model. The aim of the investigation was a comparison between microemulsion and polymer-enriched microemulsion with carbamazepine. Both formulations were radiolabeled and administered to Wistar rats. The drug concentrations in the brain observed after intranasal administration of microemulsion and mucoadhesive microemulsion were significantly higher than those recorded in the case of intravenously administered microemulsion, which can be attributed to direct nose-to-brain drug delivery. Significantly lower concentrations in brain tissue were also observed for carbamazepine solution administered intranasally. The observed effect confirms permeation enhancing potential of microemulsion carrier. It is also noteworthy that in the case of mucoadhesive microemulsion the highest concentrations were observed. The visualization of the drug distribution after intranasal and intravenous administration obtained with gamma scintigraphy camera is as shown in Figure 5. As a consequence of the observed effects, decrease in dose and dosing frequency could presumably achieve the desired therapeutic effect. It should be also emphasized that with intranasal administration of the drugs, unwanted peripheral tissue distribution of the drugs and hence the associated peripheral side effects could be avoided.

Phenytoin is a hydantoin derivative that exhibits voltage-dependent sodium channel-blocking activity. Despite its high effectiveness in long term treatment of tonic-clonic and partial seizures, it has several drawbacks such as poor solubility in water and, as a result, low absorption from the gastrointestinal tract. Another factor limiting its efficacy is an extensive hepatic metabolism. Moreover, it causes several neurological and circulatory side effects and because of its narrow therapeutic index the pharmacotherapy with phenytoin has to be monitored [158]. In the study presented by Acharya et al. [159], a phenytoin-loaded microemulsion was prepared with Capmul^®^ MCM as an oil phase and Labrasol^®^/Transcutol^®^ mixture as surfactant/co-surfactant system. The obtained microemulsion exhibited no toxicity towards the sheep nasal mucosa and was physically stable during three months of storage. An in vivo brain uptake study and gamma scintigraphy imaging performed with animal model revealed better results after intranasal microemulsion administration compared to intraperitoneal solution administration. Gamma scintigraphy images obtained for intranasal system showed the presence of drug in the brain tissue and respiratory tract, while after intraperitoneal administration, only small amount of phenytoin was transported to the central nervous system and accumulation in liver and spleen was observed. Intranasal microemulsion-based formulation was also more effective than oral formulation, allowing for faster recovery after epileptic seizure. Similar conclusions were drawn in the studies describing other antiepileptic agents, like carbamazepine [154,155,157] and clobazam [146]. The basic information on the mentioned studies related to antiepileptic agents incorporated in intranasal microemulsions is summarized in Table 2.

### 4.3. Schizophrenia

Schizophrenia is a chronic psychiatric disorder affecting over 20 million people worldwide [131]. The typical symptoms can be categorized as positive, like hallucinations and delusions, negative, like disruption of emotions and motivation, and cognitive ones. Patients suffering from schizophrenia experience memory impairment and attention deficit. Moreover, it affects social functioning and occupational performance [160,161]. An important concern in schizophrenia management is poor patient compliance, which may affect long-term outcomes [162]. The exact mechanisms underlying the disease are not understood yet. The most important hypothesis formulated to explain the occurrence of schizophrenia is related to abnormalities in dopaminergic activity in mesolimbic and mesocortical pathways [161]. Other hypotheses indicate the importance of inflammatory processes resulting from complex interaction of genetic factors, trauma, stress, and other elements. Other theories include the dysfunction in glutamergic and α7-acetylcholine neurotransmission, as well as hormone deficiency and alterations in endogenous cannabinoid system [161]. The most common therapeutic approach in schizophrenia management is focused on the interaction with dopamine receptors. It is noteworthy that currently applied antipsychotic drugs have numerous drawbacks related to their severe side effects including weight gain, sedation, extrapyramidal and other effects. The adverse drug reactions accompanying the therapy may contribute to the reduction of patient compliance [163]. Nose-to-brain formulations can be an interesting alternative to oral therapy, providing direct transport of the active ingredient to the brain tissue. In this way, the hepatic first pass effect can be avoided. Some of the observed side effects are resulting from the interaction of the drugs with peripheral dopaminergic receptors, which can be eliminated by the direct transport to central nervous system [164]. Most antipsychotic drugs reveal poor solubility in water, and they require a carrier providing sufficient concentration in nasal cavity. Microemulsions seem to be good candidates for this purpose, as they exhibit advantageous solubilizing properties for both lipophilic and hydrophilic compounds.

Patel et al. [165] developed olanzapine-loaded microemulsions for nose-to-brain delivery. Olanzapine is a commonly applied second-generation antipsychotic drug exhibiting low solubility in water and poor bioavailability related to intensive hepatic metabolism [166]. As a result, high doses are required to achieve therapeutic drug levels in the brain. Therefore, an intranasal drug delivery system can be a promising alternative to oral and parenteral formulations. In the presented study, oleic acid was used as an oil phase, and as a surfactant and co-surfactant, Kolliphor^®^ RH40 and Transcutol were applied, respectively. In order to enhance adhesion to the nasal mucosa, polycarbophil as mucoadhesive polymer was added. The obtained formulations were characterized in vivo with the use of animal models. Pharmacodynamic studies, including apomorphine-induced compulsive behavior test and spontaneous motor activity tests, were conducted with the use of mouse model. Apomorphine was used as a model dopamine receptor agonist inducing stereotyped behavior in mice. It was shown that both olanzapine-loaded microemulsion and mucoadhesive microemulsion reduced the effect related to apomorphine administration better than microemulsion administered intravenously and plain drug solution administered nasally. It is noteworthy that the observed results were better for polymer-enriched formulation, which indicates the importance of mucociliary clearance and beneficial action of mucoadhesive excipients in nasal formulations. In a spontaneous motor activity test, L-dopa and carbidopa were applied to develop schizophrenia model by increasing the concentration of dopamine in mesolimbic region. Again, mucoadhesive microemulsion-based formulation exhibited the best effects in terms of reduction of locomotor activity of the studied animals. Pharmacokinetic studies were performed with radiolabeled formulations administered to rats to check biodistribution of the drug in tissues and organs. It was shown that olanzapine concentrations in brain tissue were significantly higher after both microemulsion-based formulations administered intranasally at all time points compared to intravenous microemulsion administration and intranasal solution. Other pharmacokinetic parameters, like elimination rate constant and biological half-life, were similar regardless of the applied carrier and administration route. Radiolabeled formulations were also administered to rabbits to obtain gamma scintigraphy images showing biodistribution of applied radioisotopes. The obtained images show the highest radioactivity in brain after the administration of mucoadhesive microemulsion followed by non-mucoadhesive microemulsion. In the case of intravenous administration, the image shows peripheral distribution of radioisotope, which is not discernible in the images obtained after intranasal administration. The presented results confirm the existence of a direct nose-to-brain pathway and the show that it can be successfully applied in drug delivery.

Gadhave et al. [167] pointed to leukopenia as a possible side effect related to peripheral action of olanzapine. Intranasal nanostructured lipid carrier (NLC) and microemulsion were developed and investigated as an alternative to conventional oral formulations to avoid peripheral side effects. The investigated microemulsion system was composed of Labrafil^®^ M 1944 CS as an oil phase, Cremophor^®^ RH 40 as a surfactant, and ethanol as a co-surfactant. Additionally, hydroxypropyl methylcellulose (HPMC K4M) was applied as a mucoadhesive agent and thermosensitive poloxamer 407 was added as in situ gelling agent. An ex vivo study performed with the use of excised sheep nasal mucosa revealed higher permeation rates for olanzapine-loaded microemulsion and mucoadhesive microemulsion compared to NLC. It was also found that in both NLC and microemulsion, the presence of mucoadhesive components enhanced the permeation of the active ingredient through the mucous membrane. However, the in vivo study performed with radiolabeled samples revealed higher drug concentrations in brain after the administration of NLC-based systems compared to microemulsions. On the other hand, both nanoformulations allowed for obtaining better results than the formulation administered intravenously. The differences observed between NLC and microemulsion-based systems could be related to different viscosities of both systems. It was also found that the microemulsion-based formulation was less selective in terms of brain drug delivery and some distribution to liver, intestine, and stomach was observed. Both investigated systems were more selective than the intravenous formulation which is a promising result in terms of avoiding peripheral side effects. However, in the case of mucoadhesive microemulsions, some effects related to nasal mucosa irritation were observed.

Another atypical antipsychotic drug frequently applied in the management of schizophrenia is quetiapine. Its biological half-life is only 6 h and, as a result, frequent drug administration is necessary to achieve and maintain the therapeutic drug levels. Moreover, quetiapine shows low solubility in water and low bioavailability, which is also related to hepatic first pass metabolism. It is also a substrate for P-glycoprotein preventing its permeation across the blood–brain barrier [160,168]. Shah et al. [160] designed and developed quetiapine-loaded microemulsions with and without chitosan as permeability and bioadhesion enhancer. The authors applied Capmul^®^ MCM EP as an oil phase, Tween^®^ 80 as a surfactant, and Transcutol^®^ P as a co-surfactant. Ex vivo models were used to evaluate mucoadhesive properties of the investigated formulations, as well as drug permeation through nasal and intestinal mucosae. In the experiment with nasal mucosa, the highest permeation rate was observed in the case of chitosan-enriched microemulsion and quetiapine solution with verapamil hydrochloride applied as glycoprotein-P inhibitor. The lowest rate was recorded for plain drug solution. A similar pattern was observed in the permeation through intestinal mucous membrane. An in vivo study was performed with Sprague Dawley rats. The highest brain/blood ratio of quetiapine was observed for mucoadhesive microemulsion followed by regular microemulsion, which indicates better retention of polymer-loaded system at the administration site and improved permeability due to incorporation of the drug in microemulsion droplets.

The same research group [169] investigated microemulsions with butter oil as potential permeation enhancer in nose-to-brain delivery of quetiapine as a model drug. The composition of the microemulsion was the same as in the previous study, i.e., Capmul^®^ MCM EP was used as an oil phase, Tween^®^ 80 as a surfactant, and Transcutol^®^ P as a co-surfactant. The drug was added to microemulsion in the form of dispersion in butter oil. In ex vivo studies involving goat nasal mucosa, the highest drug flux was observed for butter oil-enriched microemulsion, followed by regular microemulsion. An in vivo study focusing on the comparison between different quetiapine-loaded formulations administered intranasally and intravenously revealed that the highest drug levels in the brain were observed at all time points for butter oil-enriched formulation administered to the nasal cavity. The highest drug levels in plasma were recorded for intranasal butter oil-enriched formulation and regular microemulsion administered intravenously.

Paliperidone is another second-generation antipsychotic indicated in the treatment of schizophrenia. It has extremely low solubility in water and low bioavailability which may cause the same problems in effective brain targeting as described for olanzapine and other neuroleptic agents. Patel et al. [170] described paliperidone-loaded microemulsion containing oleic acid as an oil phase, Cremophor^®^ RH40 as a surfactant and Transcutol^®^ as a co-surfactant. Polycarbophil was added as mucoadhesive agent. Behavioral studies including apomorphine-induced compulsive behavior test and spontaneous motor activity test revealed better effectiveness of mucoadhesive microemulsion administered intranasally compared to the regular microemulsion administered intranasally and intravenously and plain drug solution administered to the nasal cavity. The investigated formulations were tagged with radioisotopes and their effectiveness was evaluated in pharmacokinetic studies. It was shown that the drug concentrations in brain were significantly higher after intranasal administration of regular and polymer-loaded microemulsion compared to microemulsion administered intravenously. The observed effect was attributed to direct nose-to-brain transport. Moreover, gamma scintigraphy visualization indicated significantly higher radioactivity in peripheral regions after intravenous administration, while after intranasal administration radioactivity was detected mostly in central nervous system.

The same research group presented another study focusing on intranasal delivery of paliperidone-loaded microemulsion [171]. Compared to the previous one, the microemulsion system contained mixture of Cremophor^®^ RH40 and Labrasol^®^ as a surfactant system. The obtained drug delivery system was subjected to in vitro permeation study with the use of excised sheep mucosa. In the study, drug-loaded microemulsion was compared to similar system with addition of mucoadhesive polymer and to plain drug solution. It is noteworthy that no statistically significant differences were visible between the investigated drug delivery systems. The same microemulsion was analyzed as a carrier for risperidone [172]. Again, no significant differences were seen in the comparative in vitro permeation study with animal nasal mucosa.

A similar study was conducted for microemulsion with asenapine, a highly lipophilic atypical antipsychotic drug with extremely low bioavailability [173]. The investigated system contained Capmul^®^ MCM, Tween^®^ 80, and propylene glycol. In order to enhance bioadhesive properties of the obtained dispersion, polycarbophil was added. In drug diffusion performed with the use of synthetic membrane, five different samples containing different ratios of oil, water, and surfactants were analyzed. No statistically significant differences were obtained between the investigated systems. Drug permeation experiment with excised animal mucosa was conducted for one selected microemulsion with or without polycarbophil in comparison to plain drug solution. It was shown that the addition of mucoadhesive polymer enhanced drug permeation through mucous membrane.

Sulpiride is an antipsychotic agent selectively blocking dopamine receptors. It is applied in schizophrenia and also anxiety and mild depression. Similar to the previously mentioned neuroleptic agents, it reveals poor solubility in water and bioavailability. Ayoub et al. [174] developed microemulsions for nasal delivery of sulpiride. Based on the drug solubility studies, four systems with glyceryl monooleate and Labrafil^®^ as oil phases and different surfactant/co-surfactant mixtures were selected. The obtained formulations were evaluated for drug release with the use of synthetic membranes mounted in Franz diffusion cells and for drug permeation through sheep nasal mucosa. It was found that in both experiments, the drug incorporated in microemulsions diffused faster compared to plain solution used as a reference. The differences between the particular formulations related to different solubility of the drug in the applied microemulsions. Behavioral tests performed after nasal application showed that the analyzed microemulsions had an effect equivalent to sulpiride administered intravenously. However, the authors report low viscosity of microemulsions and short residence time in nasal cavity due to formulation leakage. The studies involving antipsychotic drugs administered intranasally in a form of microemulsions are summarized in Table 3.

### 4.4. Other Applications

Nose-to-brain microemulsions have also been investigated as potential carriers for the delivery of other active ingredients, including analgesic drugs. Lalani et al. [175] performed a comparative study involving tramadol-loaded intranasal microemulsion and nanoemulsion. Microemulsion contained isopropyl myristate (IPM) as on oil phase, a mixture of Labrasol^®^ and Tween^®^ 20 as surfactants, and distilled water as polar phase. Nanoemulsion was composed of IPM as an oil, soya lecithin, and poloxamer as surfactants. The investigated systems were checked for ex vivo diffusion and potential toxicity with the use of nasal sheep mucosa. Moreover, in vivo studies with the use of animal model were done to assess biodistribution of the drug, as well as pharmacokinetic and pharmacodynamic effects. It was shown that the permeation across nasal mucosa was significantly faster in the case of both emulsion-based systems compared to plain tramadol solution used as a reference. However, in the case of tramadol-loaded microemulsion, the permeation rate was higher than in the case of nanoemulsion. Toxicity studies were performed with the use of excised sheep nasal mucosa with phosphate buffer (pH = 6.4) and isopropyl alcohol used as references. It was found that the investigated microemulsion showed some toxic effects against nasal mucosa after 1h and after 2h the damage was significant with the loss of epithelial layer. No such changes were observed for the nanoemulsion formulation. Brain targeting efficiency observed after nasal administration of the investigated formulations was significantly higher compared to tramadol solution administered intranasally and intravenously. In antinociceptive effects evaluated in paw withdrawal tests, the same trend was observed.

Bshara et al. [176] investigated intranasal microemulsion-based systems with buspirone hydrochloride, partial agonist of 5-HT_1A_ serotonin receptors, and antagonist of dopamine D_2_ receptors, revealing also selective anxiolytic activity [177]. The active ingredient exhibits poor oral bioavailability related to its low permeability across biological membranes and poor absorption from gastrointestinal tract. The described effect is related to high polarity of the drug. Another factor contributing to low bioavailability of buspirone is an extensive first-pass effect. The aim of the study was to obtain a therapeutic drug level in brain tissue with the use of stable mucoadhesive buspirone-loaded microemulsion. The investigated formulations consisted of isopropyl myristate as an oil phase, Tween^®^ 80 as a surfactant, and propylene glycol as a co-surfactant. Chitosan aspartate was applied as mucoadhesive agent, while hydroxypropyl-β-cyclodextrin was applied as absorption enhancer. The obtained systems were subjected to physicochemical analyses. Mucoadhesive properties were determined as a force required for the detachment of the formulation from hydrated mucin disc. The strongest mucoadhesion was observed in the case of the formulation enhanced with both chitosan and cyclodextrin derivatives. Ex vivo experiments performed with sheep nasal mucosa mounted in modified Franz diffusion cells revealed significantly higher permeation rates for microemulsion-based systems compared to plain buspirone hydrochloride solution. Permeation rate was the highest in the case of chitosan- and cyclodextrin-enhanced system, followed by chitosan-loaded formulation and microemulsion without additives. In vivo studies performed with Wistar albino rats revealed that the highest drug concentrations in plasma were observed in the case of drug solution administered intravenously. In the case of microemulsion-based formulations administered intranasally significantly higher drug concentrations compared to drug solution administered intranasally and intravenously were observed. The enhancement effect was the highest for the formulation containing both additional components, i.e., chitosan and cyclodextrin. The histopathological analysis of the tissues exposed to the action of the investigated formulations performed after seven days of treatment revealed only some mild changes including edema and congestion of blood vessels in lamina propria. However, no signs of necrosis or hemorrhage were noted.

Another condition requiring quick and efficient drug delivery to brain is migraine. Migraine attacks are frequently accompanied with nausea and vomiting, which is a cause of insufficient drug absorption from gastrointestinal tract. In such cases, an alternative drug delivery route should be taken into consideration. The therapeutic options currently available on the pharmaceutical market include nasal sprays, e.g. Tosymra^®^ (Upsher-Smith) [178]. However, the studies aiming at the development of novel carriers for antimigraine drugs and the improvement or modification the therapeutic effect are still gaining a lot of interest [179]. Vyas et al. [180] presented investigations focused on microemulsion loaded with sumatriptan and sumatriptan succinate, serotonin agonists commonly applied in the treatment of migraine attacks. Microemulsion consisted of medium chain triglyceride (MCT) as an oil phase, caprylocaproyl macrogol glyceride as a surfactant, and Transcutol^®^/fatty acid ester of polyglycerol as a co-surfactant. In order to improve mucoadhesive properties of the obtained formulations, polycarbophil was added. The obtained formulations were radiolabeled and evaluated with Swiss albino rat model for drug biodistribution with gamma scintigraphy imaging method. Moreover, the drug levels in the brain and plasma were evaluated. It was found that the concentrations in brain were higher at all time points in the case of sumatriptan-loaded microemulsion and polycarbophil-enhanced microemulsion administered nasally compared to microemulsion administered intravenously. Intranasal microemulsion with sumatriptan showed also higher brain/blood ratio compared to plain sumatriptan solution and to commercially available product. Higher drug targeting efficiency was recorded for mucoadhesive formulation with polycarbophil, which was also observed by many other authors comparing polymer-thickened and non-thickened microemulsions. Sumatriptan succinate-loaded microemulsion with and without polycarbophil revealed similar effects to sumatriptan succinate solution and sumatriptan marketed product administered nasally in terms of direct nose-to-brain transport and drug targeting efficiency. However, the pharmacokinetic parameters, like AUC and C_max_, were higher in the case of microemulsion-loaded formulations. Moreover, the drug levels in the brain were higher in the case of microemulsion systems loaded with sumatriptan compared to the sumatriptan salt, which was related to higher lipophilicity of the neutral drug form and different mucociliary clearance of both forms. Gamma scintigraphy images taken 0.5 h after intranasal and intravenous administration of the investigated formulations revealed higher radioactivity in brain after nasal administration of mucoadhesive system with sumatriptan compared with non-mucoadhesive one applied intranasally and intravenously. Moreover, in the described study, electron micrographs of human nasal mucosa treated with sumatriptan succinate solution, sumatriptan-loaded microemulsion, and sumatriptan-loaded mucoadhesive microemulsion were presented. The obtained images indicate the dilation of tight junctions in epithelium upon the contact with microemulsion-based systems. The changes were not observed in solution-treated mucosa. It was also shown that altered tight junctions returned to their original shape after washing the mucosa, which indicated the reversibility of the processes observed as a result of microemulsion application. On the other hand, this observation suggests paracellular drug transport in the investigated systems.

Intranasal microemulsions have also been investigated as alternative drug carriers in glioblastoma, a highly aggressive malignant brain tumor with a five-year survival rate lower than 5% [181]. Standard therapeutic approach involves radio- and chemotherapy with temozolomide. As it was already mentioned, drug delivery to brain tissue is challenging and usually poor solubility of the active ingredient and its low permeability across the blood–brain barrier are the factors reducing therapeutic efficacy. Therefore, alternative treatment options are extensively investigated. Gadhave et al. [182] investigated intranasal mucoadhesive microemulsion loaded with teriflunomide, tyrosine kinase and dihydroorotate dehydrogenase inhibitor reducing biosynthesis of pyrimidine in cancer cells. The drug is highly hepatotoxic and oral administration is not recommended due to the severity of possible side effects. Moreover, it does not permeate through BBB easily. The investigated microemulsion was composed of Maisine^®^ 35-1 as an oil phase, Labrasol^®^ as a surfactant, and Transcutol^®^ HP as a co-surfactant. The addition of poloxamer 407 and hypromellose provided thermosensitive properties allowing for gelation in situ upon the physiological temperature in nasal cavity. An ex vivo permeation study conducted with sheep nasal mucosa revealed higher flux values for polymer-enhanced formulation compared to the non-modified one. Brain targeting studies performed with Swiss albino mice indicated selective transport of active ingredient to the brain tissue without crossing BBB. As it was shown in many other studies, mucoadhesive formulation revealed better ability to deliver the drug to central nervous system. However, no other formulations were used as a reference. Histopathological and hematological tests showed no significant abnormalities, except for the slight erosion of nasal tissues related to the administration of high doses of teriflunomide. The obtained results are promising in terms of side effects reduction and better brain targeting.

Another study aiming at the formulation of novel drug delivery system for the treatment of glioblastoma was presented by Mena-Hernández et al. [183]. As an active ingredient, mebendazole, a commonly known antihelmintic agent with proven antiproliferative activity, was applied. The drug is poorly absorbed from gastrointestinal tract due to low solubility in water. Moreover, mebendazole is extensively metabolized by hepatic enzymes. The investigated microemulsion consisted of oleic acid combined with docosahexanoic acid-rich oil (DHA) and Labrafil^®^ M 2125 as an oil phase, Tween^®^ 80 as a surfactant, Transcutol^®^ HP as a co-surfactant, and ethanol as a co-solvent. As a mucoadhesive agent, sodium hyaluronate was applied. In the in vivo study, male Wistar rats with implanted C6 rat glioma cell line were used. The animals were treated with mebendazole-loaded mucoadhesive microemulsion compared to corresponding placebo formulation. The first group survived the full duration of the experiment (50 days) while the placebo group survived only 20–30 days. Another study involved fluorescence imaging of integrins, cell surface glycoproteins with intensive expression in glioma tumors. The applied technique showed the increased fluorescence signal after 7 days from tumor cells implantation. The studies performed after the treatment with mebendazole-loaded formulations revealed that the signal was less intense in the group treated with the active ingredient which indicated the reduction of tumor size. However, some reduction in placebo group was also observed. It was also noted that the tissue samples obtained from mebendazole-treated group contained less features typical for malignant tumors.

## 5. Conclusions and Future Directions

Nose-to-brain drug delivery attracts enormous attention as an alternative to conventional therapeutic approaches. Among the most important advantages of intranasal formulations are ease of administration, rapid onset of action, selective drug delivery to brain tissue, and the possibility to avoid peripheral effects. However, intranasal drug delivery has also several drawbacks. One of them is related to small volume of the nasal cavity limiting the amount of the formulation that can be administered to the nasal mucosa. Moreover, nasal cavity is anatomically and physiologically predisposed to remove potentially harmful exogenous substances, including drugs; and mucociliary clearance and poor permeation through nasal mucous membrane are frequently mentioned as challenges in nose-to-brain drug delivery. All of the mentioned issues can be addressed with the use of proper drug carrier exhibiting sufficient solubilizing properties and permeation enhancing ability. In this way, high concentration of the active ingredient in the applied formulation allows for the reduction of the applied formulation volume. Microemulsions are commonly known for their ability to incorporate relatively high amount of active ingredients revealing various polarities and to increase the permeation through various biological membranes. The available literature reports mentioned in this review indicate that these systems can also be useful in direct drug delivery to brain via nasal route. In all in vivo studies involving animal models, significant improvement in terms of drug amounts delivered to the brain tissue and brain targeting were observed. The obtained results are particularly valuable for the research area related to the diseases managed with the use of active ingredients causing severe side effects associated with drug distribution to peripheral organs and tissues. The comparisons made between intranasal microemulsion-based formulations and equivalent drug delivery systems administered parenterally show that intranasal administration provide quick and efficient brain targeting, which confirms the existence of nose-to-brain pathway. However, all of the available studies were performed with the use of animal models and the anatomical and physiological differences between species are obvious. It is noteworthy that no human studies with the use of microemulsion-based carriers have been performed yet, even though some clinically relevant studies related to nose-to-brain delivery in general are available [117,184]. Some of the available literature reports referring to humans indicate no additional uptake of the active ingredient in the central nervous system [185,186], and some focusing on the same active ingredient provide contradictory results [184,186]. Therefore, the efficacy and safety of intranasal microemulsions designed for human brain targeting require further investigations. The same issue has been raised for other nanodispersions evaluated as potential nose-to-brain drug delivery systems, including nanoemulsions [36,187,188]. It should also be noted that some of the studies mentioned in this review present ex vivo experiments only, which provide valuable information on the interaction between the formulation and nasal mucosa but are insufficient to draw any conclusions on the clinical performance of the analyzed systems.

Another important question requiring further analyses is the efficacy of microemulsion-based systems for nose-to-brain drug delivery in comparison to other nanodispersions, like nanoparticles, nanoemulsions, or micelles, applied as drug carriers. So far, only two comparative studies describing the performance of microemulsion compared to nanostructured lipid carrier and nanoemulsion were presented by Gadhave et al. [167] and Lalani et al. [175], respectively. The results obtained in the first study showed that the microemulsion was less promising than NLCs in terms of potential therapeutic usefulness. In the other one, slightly better pharmacokinetic properties and slightly increased ciliotoxicity were observed in the case of microemulsion. However, further investigations are necessary to gain better insight into the most efficient methods to utilize the potential of nose-to-brain pathways. The presented studies usually do not evaluate the effects of different oils, surfactants and co-surfactants on pharmacokinetic parameters of the analyzed formulations, except for the studies focusing on butter and fish oils as potential permeation enhancers [138,139,169]. The mentioned studies show that the modifications in microemulsion composition can significantly change the permeability of nasal mucosa which may well affect the therapeutic efficacy of the applied formulation. However, the literature reports regarding this issue are still scarce. At the same time, numerous studies present the importance of viscosity modifying agents added to microemulsions to enhance their mucoadhesive properties. It is to be emphasized that microemulsions due to low viscosity are susceptible to easy removal from the nasal cavity as a result of mucocilliary clearance. It was shown that the addition of polymers like chitosan or polycarbophil extends the residence time at the administration site and improves the efficacy of the formulation. Moreover, according to Shah et al. [42], chitosan exhibits the ability to modify the tight junctions in nasal epithelium, which is important for enhancing the paracellular transport of active ingredients. As a result, more promising results were obtained for the polymer-enriched formulations than for non-modified equivalents.

In the presented studies, the toxicity of microemulsions towards nasal mucosa was also analyzed. It must be highlighted that surfactants and co-surfactants are necessary to formulate microemulsions but can be potentially harmful and cause irritation of mucous membrane [136]. The same concern has been described for nanoemulsions, which are usually composed of similar ingredients. According to Bonferoni et al. [36], simplified toxicity studies presented for these systems are insufficient for proper safety evaluation in the case of the formulations administered repeatedly which is usually required in chronical illnesses. It must be emphasized that microemulsions usually contain higher amounts of surface-active agents, which increase the risk of side effects and ciliotoxicity. According to the presented studies, usually no evidence of irritation was shown for the investigated microemulsions, except for the study presented by Lalani et al. [175]. However, in future investigations the toxicity of microemulsions administered intranasally in long-term therapies should be analyzed in detail. Contradictory results regarding the toxicity of the investigated systems suggest that the side effects may depend on the exact composition and concentrations of particular components in microemulsions. Therefore, the excipients selection should be carefully considered with special attention paid to less-irritating components and possibly lower surfactant concentrations. The comparative study presented by Lalani et al. [175] indicates that similar results may be achieved with nanoemulsions, which usually contain lower amounts of potentially harmful ingredients. However, as it was already mentioned, more research is necessary to assess safety of intranasal microemulsion administration.

## Figures and Tables

**Table 1:** Microemulsion-based systems investigated in nose-to-brain delivery in neurodegenerative diseases.

Active Component	Microemulsion Components	Drug Release/Permeation Assessment	General Conclusions	References
rivastigmine hydrogen tartrate	Capmul^®^ MCM EP, Labrasol^®^, Transcutol^®^ P, water, chitosan, cetyltrimethylammonium bromide	in vitro: Franz cells, cellulose acetate membrane (m.w. cut-off 12,000–14,000) ex vivo: Franz cells, goat nasal mucosa	chitosan-based microemulsion showed improved ex vivo permeation	[136]
rivastigmine hydrogen tartrate	Capmul^®^ MCM EP, Labrasol^®^, Transcutol^®^ P, water, chitosan	in vitro: Franz cells, cellulose acetate membrane in vivo: male Sprague-Dawley rats, blood and brain concentration, gamma scintigraphy visualization	addition of chitosan contributed to higher brain concentration of the drug	[42]
rivastigmine hydrogen tartrate	Capmul^®^ MCM EP, Labrasol^®^, Transcutol^®^ P, water, butter oil, fish oil	in vitro: evaluation of the protective role of ME against Amyloid Beta (1–42) oligomer induced toxicity in IMR 32 cell line	fish oil and butter oil acted as penetration enhancers through nasal mucosa, no protection in IMR 32 cell line	[137]
galantamine hydrochloride	Capmul^®^ MCM EP, Labrasol^®^, Transcutol^®^ P, water, butter oil, fish oil	ex vivo: Franz cells, goat nasal mucosa	enhancement of permeation by addition of fish and butter oils	[138]
donepezil hydrochloride	Capmul^®^ MCM EP, Tween^®^ 20, Transcutol^®^ EP, water, butter oil, omega-3 fish oil	ex vivo: Franz cells, goat nasal mucosa in vitro: cell permeability studies on bEnd.3 mouse cerebral microvascular endothelial cell line	fish oil induced higher bioavailability than butter oil	[139]
tacrine	Labrafil^®^ M 1944 CS, Cremophor^®^ RH 40, Transcutol^®^ P, water	in vivo: male C57BL/6 mice, intranasal administration, ventral mid brain and striatum drug concentration, behavioral tests	in scopolamine-induced amnesia model in mice the fastest recovery was reached for microemulsions	[140]
donepezil hydrochloride	castor oil, Labrasol^®^, Transcutol^®^ P, propylene glycol	in vitro: Franz cells, dialysis membrane (pore size 12–14 kDa) ex vivo: Franz cells, porcine nasal mucosa	more than 32% of the drug retained in porcine nasal mucosa	[141]
huperzine A	1,2-propanediol, castor oil Cremophor^®^ RH40, water, Pluronic F68, chitosan	in vitro: Franz cells, dialysis membrane (m.w. cut-off 6000–8000 U) in vivo: male Sprague-Dawley rats, microdialysis assay	after nasal administration both the plasma and brain concentration profiles showed the evidence of sustained and prolonged release, also higher bioavailability was observed	[142]
morin hydrate	Capmul^®^ MCM, Cremophor^®^ EL, PEG-400, water	in vitro: Franz cells, cellulose membranę behavioral tests	significant memory improvement in rats with streptozotocin-induced dementia	[143]
vinpocetine, piracetam	Tween^®^ 20, oleic acid, ethanol, water, soybean lecithin—Epikuron^®^ 200	in vivo: male Wistar rats, brain drug concentration determination, behavioral tests	increase of both pharmaceutical and pharmacological properties due to application of nanocarriers	[144]
ibuprofen	Capmul^®^ MCM, Accenon^®^ CC, Transcutol^®^, water, polycarbophil	in vitro: Franz cells with sheep mucosa in vivo: male C57BL/6 mice, striatal dopamine concentrations, behavioral tests, nasal cilitoxicity	increased dopamine levels and better motor activity due to application of ibuprofen-loaded microemulsion, no toxicity	[145]

**Table 2:** Microemulsion-based systems investigated in nose-to-brain delivery in epilepsy.

Active Component	Microemulsion Components	Drug release/Permeation Assessment	General Conclusions	References
clobazam	Capmul^®^ MCM, Acconan^®^ C6, Tween^®^ 20, water, Carbopol 940P	ex vivo animal mucosa, in vivo gamma-scintigraphy, pharmacodynamic tests	better efficacy of mucoadhesive formulationintranasal system	[146]
lorazepam	Capmul^®^ MCM, Nikkol PBC-34, Transcutol^®^ P, water, gellan gum, Carbopol^®^	ex vivo goat nasal mucosa, pharmacodynamic tests (including behavioral ones)	faster and longer duration of action than the marketed product; better results for mucoadhesive formulation	[152]
diazepam	oleic acid, Tween^®^ 80, propylene glycol, water, chitosan	in vivo pharmacokinetic studies, behavioral tests	enhanced brain delivery in microemulsion systems; better performance of mucoadhesive product	[153]
carbamazepine	oleic acid, Tween^®^ 80, propylene glycol or Transcutol^®^, water	ex vivo sheep nasal mucosa, in vivo pharmacokinetic studies, induced convulsions in mice	seizure time reduction similar to intraperitoneal drug solution; higher drug concentration in brain tissue for Transcutol^®^-based microemulsion	[154,155]
carbamazepine	Labrafil^®^ M1944, Cremophor^®^ RH40, Transcutol^®^, water, polycarbophil	ex vivo sheep nasal mucosa, pharmacokinetic studies, gamma scintigraphy	no significant differences between microemulsion-based systems and drug solution in ex vivo study; higher concentrations in brain obtained for microemulsions; selective accumulation in brain	[156,157]
phenytoin	Capmul^®^ MCM, Labrasol^®^, Transcutol^®^, water	in vivo pharmacokinetic studies, gamma scintigraphy, induced convulsions in mice	better selectivity towards brain compared to intraperitoneal administration; faster recovery after epileptic seizure	[159]

**Table 3:** Microemulsion-based systems investigated in nose-to-brain delivery in schizophrenia.

Active Component	Microemulsion Components	Drug Release/Permeation Assessment	General Conclusions	References
olanzapine	oleic acid, Kolliphor^®^ RH40, Transcutol^®^, water, polycarbophil	in vivo pharmacokinetic studies; pharmacodynamic tests; gamma scintigraphy	higher concentration in brain compared to intravenous microemulsion and intranasal solution; no peripheral distribution	[165]
olanzapine	Labrafil^®^ M1944CS, Cremophor^®^ RH40, ethanol, water, HPMC K4M, poloxamer 407	ex vivo sheep nasal mucosa; in vivo studies; gamma scintigraphy	higher permeation rate compared to NLC; lower drug concentrations in brain compared to NLC; less selective drug delivery than NLC; nasal mucosa irritation	[167]
quetiapine	Capmul^®^ MCM EP, Tween^®^ 80, Transcutol^®^ P, water, chitosan	ex vivo nasal and intestinal mucosa; in vivo pharmacokinetic studies	the highest permeation rate ex vivo and the highest drug level in brain in vivo was observed for chitosan-loaded microemulsion	[160]
quetiapine	Capmul^®^ MCM EP, Tween^®^ 80, Transcutol^®^ P, water, butter oil	ex vivo goat nasal mucosa; in vivo pharmacokinetic studies	the highest permeation rate ex vivo and drug levels in plasma were observed for butter oil-enriched microemulsion	[169]
paliperidone	oleic acid, Cremophor^®^ RH40, Transcutol^®^, water, polycarbophil	behavioral studies, pharmacokinetic in vivo studies, gamma scintigraphy	mucoadhesive microemulsion exhibited the best performance in behavioral studies and the better selectivity than intravenous formulation	[170]
paliperidone	oleic acid, Cremophor^®^ RH40, Labrasol^®^, Transcutol^®^, water, polycarbophil	ex vivo sheep mucosa	no significant differences between microemulsion, mucoadhesive microemulsions and drug solution	[171]
risperidone	oleic acid, Cremophor^®^ RH40, Labrasol^®^, Transcutol^®^, water, polycarbophil	ex vivo sheep mucosa	no significant differences between microemulsion, mucoadhesive microemulsions and drug solution	[172]
asenapine	Capmul MCM, Tween 80, propylene glycol, water, polycarbophil	drug release with synthetic membrane, drug permeation with excised animal mucosa	no significant differences between samples with different composition in drug release study; permeation through nasal mucosa was faster for mucoadhesive formulation	[173]
sulpiride	glyceryl monooleate/Labrafil, different surfactants and co-surfactants	drug release with synthetic membranes; drug permeation through sheep nasal mucosa; behavioral tests	the differences in drug release were related to drug solubility; the same results in behavioral tests obtained for microemulsions and intravenous formulation	[174]
